# Molecular Epidemiology of *Plasmodium falciparum* Malaria Outbreak, Tumbes, Peru, 2010–2012

**DOI:** 10.3201/eid2105.141427

**Published:** 2015-05

**Authors:** G. Christian Baldeviano, Sheila Akinyi Okoth, Nancy Arrospide, Rommell V. Gonzalez, Juan F. Sánchez, Silvia Macedo, Silvia Conde, L. Lorena Tapia, Carola Salas, Dionicia Gamboa, Yeni Herrera, Kimberly A. Edgel, Venkatachalam Udhayakumar, Andrés G. Lescano

**Affiliations:** US Naval Medical Research Unit No. 6, Callao, Peru (G.C. Baldeviano, J.F. Sánchez, S. Macedo, L.L. Tapia, C. Salas, K.A. Edgel, A.G. Lescano);; Centers for Disease Control and Prevention, Atlanta, Georgia, USA (S. Akinyi Okoth, V. Udhayakumar);; Instituto Nacional de Salud del Peru, Lima, Peru (N. Arrospide); Direccion Regional de Salud de Tumbes, Tumbes, Peru (R.V. Gonzalez, S. Conde);; Universidad Peruana Cayetano Heredia, Lima (D. Gamboa); Ministry of Health of Peru, Lima (Y. Herrera)

**Keywords:** Falciparum malaria, *Plasmodium*, Peru, microsatellite markers, parasites, malaria, Peru

## Abstract

During 2010–2012, an outbreak of 210 cases of malaria occurred in Tumbes, in the northern coast of Peru, where no *Plasmodium falciparum* malaria case had been reported since 2006. To identify the source of the parasite causing this outbreak, we conducted a molecular epidemiology investigation. Microsatellite typing showed an identical genotype in all 54 available isolates. This genotype was also identical to that of parasites isolated in 2010 in the Loreto region of the Peruvian Amazon and closely related to clonet B, a parasite lineage previously reported in the Amazon during 1998–2000. These findings are consistent with travel history of index case-patients. DNA sequencing revealed mutations in the *Pfdhfr, Pfdhps, Pfcrt*, and *Pfmdr1* loci, which are strongly associated with resistance to chloroquine and sulfadoxine/pyrimethamine, and deletion of the *Pfhrp2* gene. These results highlight the need for timely molecular epidemiology investigations to trace the parasite source during malaria reintroduction events.

## Abstract 2

Multidrug-resistant parasites from the Amazon region caused the outbreak in the northern coastal region.

During the past decade, remarkable progress in malaria control has been achieved globally (*1*). As low-risk areas progress toward the preelimination phase of malaria elimination (http://www.who.int/malaria/areas/elimination/overview/en/), new challenges are posed by risk for reintroduction of parasites into areas where malaria transmission was interrupted (*2*). Human movement from malaria-endemic regions could facilitate outbreaks in areas where malaria had been eliminated (*2*,*3*). Molecular epidemiology tools have been used to investigate the sources of malaria reintroduction (*4*,*5*). Use of these tools enables rapid characterization of potentially pathogenic or multidrug-resistant strains before they become adapted and expand to other non–malaria-endemic areas where anopheline vectors are present (*6*–*9*).

In Peru, malaria reemerged in the 1990s and the number of cases peaked at ≈160,000 cases in 1998 (*10*). Most reported cases had occurred in the Amazon Basin (Loreto region) and areas in the northern Pacific coast of Peru, including the Tumbes and Piura regions. In vivo efficacy studies conducted during 1998–2000 revealed different patterns of drug resistance between parasites in the Amazon region and coastal areas (*11*,*12*). Although parasites from the eastern Amazon region were resistant to chloroquine and sulfadoxine/pyrimethamine, parasites from the northern Pacific coast were resistant to chloroquine but remained sensitive to sulfadoxine/pyrimethamine (*11*–*13*). In 2001, artesunate/mefloquine combination therapy was introduced in the Peruvian Amazon while artesunate–sulfadoxine/pyrimethamine remained in use in the northern Pacific coastal region (*12*).

After 2005, changes in drug policy and increased vector control efforts in Peru led to a drastic reduction in the number of malaria cases in the country. A major accomplishment was the interruption of *Plasmodium falciparum* transmission in the northern Pacific coast; no autochthonous malaria case has been reported since 2006. However, in October of 2010, the Regional Health Directorate in Tumbes received reports of 2 cases of *P. falciparum* malaria. An outbreak investigation confirmed the *P. falciparum* malaria epidemic in Tumbes. This outbreak continued to spread through 2012, when the last case of *P. falciparum* malaria was reported. Epidemiology investigations identified 2 index case-patients among military personnel stationed in Tumbes; surveillance activities conducted during the outbreak investigation suggested that these patients potentially acquired *P. falciparum* infection while in the Peruvian Amazon. We therefore hypothesized that a detailed genetic characterization of the parasite populations isolated during this outbreak might provide a better understanding of the source and main biological features of the parasite responsible for the reintroduction of malaria into Tumbes.

Previous genetic analyses of *P. falciparum* strains collected at the peak of the malaria epidemic, 1999–2000, revealed at least 5 distinct clonal lineages (clonets A–E), as defined by genotyping of 7 neutral microsatellite loci (*14*). These clonets, which were distributed in different areas of Peru, exhibited distinct patterns of mutations based on sequencing of the *Pfcrt, Pfmdr1, Pfdhps*, *and Pfdhfr* genes (*14*). Considering these historical data, we tested the following hypotheses. First, if the *P. falciparum* outbreak in Tumbes was caused by bottlenecked parasites from the coastal region, the parasites causing this outbreak would be genetically similar or closely related to clonet E, which was the only lineage found in the northern Pacific coast during 1999–2000. Second, if the parasite was introduced from the Peruvian Amazon, then the parasites causing this outbreak would be related to clonets A, B, C, or D. Third, if these parasites were introduced from outside Peru, they may have different molecular signatures.

## Materials and Methods

### Study Area and Sample Collection

**Figure 1:**
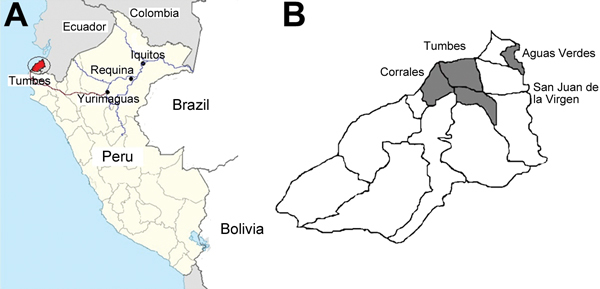
Peru, showing the department of Tumbes (black shading), the city of Iquitos, and the Requena district located in the Loreto region of the Peruvian Amazon (A) and the 13 districts in the department of Tumbes (B). Gray shading indicates the 4 districts (Tumbes, Corrales, Aguas Verdes, and San Juan de la Virgen) where the 210 cases were reported during the 2010–2012 outbreak of *Plasmodium falciparum* malaria; blue lines indicate travel routes by river; red line indicates travel route by road**.**

The Tumbes region is located in the Pacific northwestern part of Peru near the border with Ecuador (Figure 1, panel A). Tumbes is divided into 13 districts with a total surface of 4,670 km^2^ and a population of ≈228,227 (Figure 1, panel B). In the late 1990s, malaria transmitted by *P. vivax* and *P. falciparum* was highly endemic to the region (*11*,*15*). In Tumbes, *Anopheles albimanus* mosquitoes predominate*,* unlike in the Amazon region, where *An. darlingi* mosquitoes predominate. In the 2000s, malaria incidence was drastically reduced, and the parasite predominance shifted to *P. vivax*, which is seasonal in this area, peaking during the rainy season (February–June). The last autochthonous case of *P. falciparum* malaria in Tumbes was reported in 2006. 

In October 2010, after the Regional Health Directorate received reports of 2 cases of *P. falciparum* malaria, an outbreak response team led by the Ministry of Health of Peru with the support of the US Naval Medical Research Unit No. 6 (NAMRU-6), the US Centers for Disease Control and Prevention (CDC), and the US Agency for International Development conducted an outbreak investigation and response. As part of these activities, malaria cases were detected by passive surveillance of febrile patients seeking treatment at local health facilities or at the regional referral hospital. Additional cases were detected by various case-finding activities conducted in areas where laboratory-confirmed *P. falciparum* malaria cases were found. For all patients, thick and thin smears stained with Giemsa 10% were examined for parasites. Slides were read at the local health facility and sent to the National Institute of Health in Peru (INS) and NAMRU-6 for species confirmation and quality control. Blood was spotted onto Whatman 3MM filter paper (GE Healthcare, Atlanta, GA, USA) and sent to INS and NAMRU-6. For some cases, whole blood was collected by venipuncture and shipped to INS and NAMRU-6. All biological samples were collected exclusively for the purpose of diagnosis, case investigation, and patient management as part of a public health intervention led by the Ministry of Health of Peru.

### DNA Isolation and PCR Analysis

DNA was isolated from filter paper blood spots or whole blood samples by use of the QIAamp DNA Blood Mini Kit (QIAGEN, Valencia, CA, USA) as described elsewhere (*16*). Nested PCR was used to confirm *P. falciparum* infection for all patients in this study (*17*). Molecular analysis was performed at the NAMRU-6 laboratory in Lima, Peru. Selected samples were sent to CDC for further genetic characterization.

### Microsatellite Analysis

Whole-genome amplified DNA (REPLI-g; QIAGEN) was used for microsatellite characterization. All *P. falciparum*–confirmed samples were assayed for 7 putatively neutral microsatellite loci. In studies in South America, 5 microsatellite loci have been used: TA1 (chromosome 6); poly α (chromosome 4); PfPK2 (chromosome 12); TA109 (chromosome 6), and 2490 (chromosome 10) (*18*–*20*). In addition to these markers, we also amplified the loci C2M34 (chromosome 2) and C3M69 (chromosome 3) (*21*). Primer sequences and PCR parameters for these loci have been described (*14*,*16*). Furthermore, all samples were assayed for 5 microsatellite loci that span ≈11 kb on chromosome 4 around *Pfdhfr*, 9 loci that span 17 kb on chromosome 8 around *Pfdhps*, 4 loci that span 11 kb on chromosome 7 around *Pfcrt*, and 6 loci that span 8 kb on chromosome 5 around *Pfmdr1*. The primers used to amplify microsatellite loci have been described (*16*,*22*). The amplification products were labeled with fluorescent dyes (HEX or FAM) and assayed for size on an 3130xl sequencer (Applied Biosystems, Foster City, CA, USA). The fragments were then scored by using GeneMapper software version 3.7 (Applied Biosystems) with default microsatellite settings, whereby bands of <500 relative fluorescence units were defined as background. Samples for which we obtained no amplification in some loci were reanalyzed to complete the haplotypes. Earlier, we had created haplotype identifiers for each of these genes on the basis of microsatellite loci that were nearby (*16*).

### Genotyping of Markers of Drug Resistance

DNA isolates were sequenced for point mutations in *Pfdhfr*, *Pfdhps*, chloroquine resistance transporter (*Pfcrt*), and *Pfmdr1*. The methods used are described elsewhere (*14*,*16*,*21*,*23*,*24*).

### Detection of *P. falciparum* Histidine-Rich Protein 2 Deletions

Two sets of primers were designed to amplify a 228-bp fragment of *P. falciparum* histidine-rich protein 2 (*Pfhrp2*) in a nested PCR. The outward forward primer was 5′-GGTTTCCTTCTCAAAAAATAAAG-3′, and the outward reverse primer was 5′-TCTACATGTGCTTGAGTTTCG-3′. The secondary reaction used 5′-GTATTATCCGCTGCCGTTTTTGCC-3′ (forward) and 5′-CTACACAAGTTATTATTAAATGCGGAA-3′ (reverse) primers. The cycling conditions were as follows: primary reaction at 95°C for 5 min; 30 cycles of 95°C for 30 s, 60°C for 30 s, 68°C for 30 s; and 68°C for 5 min; and secondary reaction at 95°C for 5 min; 30 cycles of 95°C for 30 s, 65°C for 30 s, 68°C for 30 s; and 68°C for 5 min.

### Ethics Considerations

The activities were conducted in compliance with all applicable federal and international regulations governing the protection of human subjects. No informed consent was requested from the patients because all biological samples were collected as part of a public health intervention led by the Ministry of Health of Peru. All samples received by INS, CDC, or NAMRU-6 were coded, and no access to personal identifiable data was provided.

## Results

**Figure 2:**
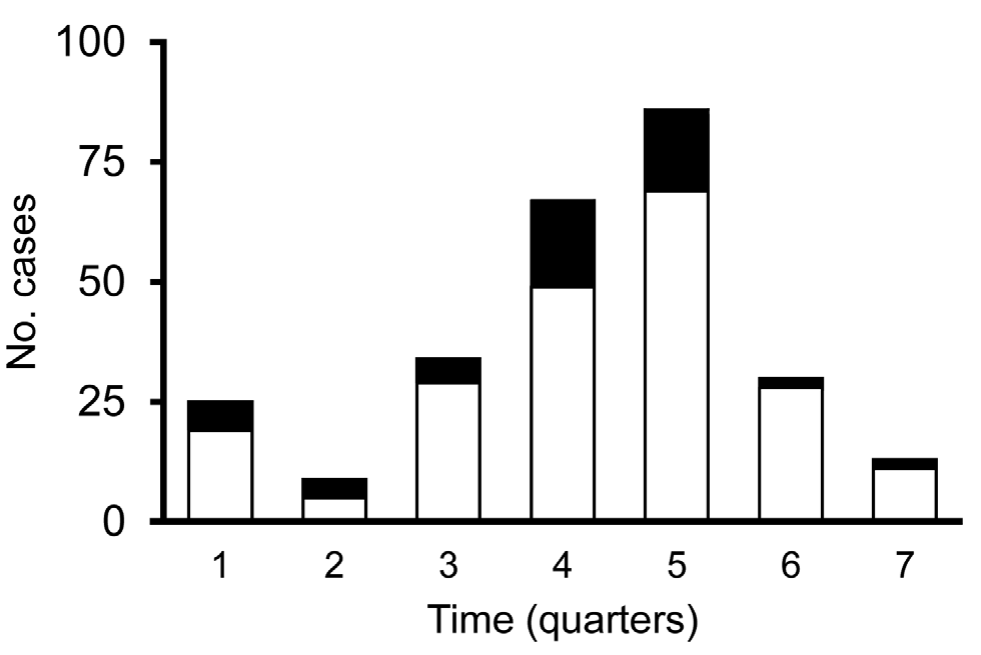
Temporal distribution of cases of falciparum malaria reported to the Tumbes Regional Health Directorate, Peru, October 2010–June 2012. Black bar sections indicate number of cases with samples available for analysis.

During October 2010–June 2012, a total of 210 cases of *P. falciparum* malaria were reported in Tumbes. Filter paper or whole blood samples were available for laboratory testing for 57 (27%) of the 210 patients. Figure 2 shows the temporal distribution of all 210 cases reported during the outbreak and the 57 cases that were included in this study. PCR confirmed *P. falciparum* monoinfection in 54 patients; *P. vivax*/*P. falciparum* mixed infections in the other 3 patients led to their exclusion from further analysis. To determine the clonal composition of the isolates, we genotyped 7 neutral microsatellite markers located in 6 different chromosomes. We observed virtually the same clonal lineage across all 54 isolates with the exception of 3 isolates that had alleles that were 3 bp shorter in loci TA1 and PfPK2 (Table 1). These results suggested that the outbreak originated from a single parasite source or from various sources of the same parasite population.

**Table 1:** Genetic lineage based on 7 neutral microsatellite markers of representative *Plasmodium falciparum* isolates from malaria outbreak in Tumbes and Amazon Region, Peru, 2010–2012*

Code	Collection Date	Isolation area	Genetic lineage	Chromosome location, locus name						
				6	4	7	6	10	2	3
				TA1	Polyα	PfPK2	TA109	2490	C2M34	C3M69
MIS 0760	2010 Oct 25	Tumbes	B_V1_	172	183	172	164	84	232	134
MIS 0761	201 Oct 26	Tumbes	B_V1_	172	183	172	164	84	232	134
MIS 1142	2011 Mar 31	Tumbes	B_V1_	172	183	172	164	84	232	134
MIS 1147	2011 Jul 19	Tumbes	B_V1_	172	183	172	164	84	232	134
MIS 1146	2011 Jul 26	Tumbes	B_V1_	172	183	172	164	84	232	134
MIS 1168	2011 Aug 19	Tumbes	B_V1_	172	183	172	164	84	232	134
MIS1280	2011 Aug 27	Tumbes	B_V1_	172	183	172	164	84	232	134
TI-435	2011 Sep 22	Tumbes	B_V1_	172	183	172	164	84	232	134
TIA-4156	2011 Sep 28	Tumbes	B_V1_	169	183	172	164	84	232	134
T3–861	2011 Oct 4	Tumbes	B_V1_	169	183	169	164	84	232	134
TIA-4333	2011 Oct 14	Tumbes	B_V1_	172	183	172	164	84	232	134
MIS1278	2011 Nov 1	Tumbes	B_V1_	172	183	172	164	84	232	134
T3–989	2011 Nov 18	Tumbes	B_V1_	172	183	172	164	84	232	134
M01L1615	2011 Dec 1	Tumbes	B_V1_	169	183	172	164	84	232	134
TIA-434	2011 Dec 1	Tumbes	B_V1_	172	183	172	164	84	232	134
PSSI-205	2011 Dec 27	Corrales	B_V1_	172	183	172	164	84	232	134
Z1–22	2012 Mar 14	Zarumilla	B_V1_	172	183	ND	164	84	232	134
PR-12†	Dec 2009	Requena (C)	B_V1_	172	183	172	164	84	232	134
PY013†	Mar 2010	Yurimaguas (E)	B_V1_	172	183	172	160	84	232	134
Clonet B†	1999–2000	Loreto (E)	B	172	183	172	164	84	226	149
Clonet A†	1999–2000	Loreto (E)	A	169	172	166	164	84	240	132
Clonet C†	1999–2000	Loreto (E, W)	C	178	164	163	160	80	246	136
Clonet D†	1999–2000	Loreto (E, W)	D	178	161	175	160	80	233	122
Clonet E†	1999–2000	Tumbes	E	172	148	175	160	74	226	138

*Microsatellite genotypes of all 11 isolates from Requena and 1 of 12 isolates from Yurimaguas were identical to those of the Tumbes outbreak. Only 17 of 54 isolates processed are shown. Isolates were selected to cover the extent of the duration of the outbreak (October 2010–March 2012). ND, not determined. †Historical microsatellite alleles of clonets A–E found in Peru in 1999–2000 and the B_V1_ variant found in Loreto in 2009–2010. C, Central Amazon; E, eastern Amazon; W, western Amazon.

In a previous study, we found 5 distinct clonal lineages (clonets A–E) among *P. falciparum* isolates collected in the Peruvian Amazon (*14*). We compared the genotype of the outbreak parasite to genotypes in a historical microsatellite database and found that the genotype of the isolates from Tumbes closely resembled clonet B, a lineage found in the eastern Peruvian Amazon during 1999–2006 (*14*). However, the outbreak haplotype differed at 2 loci: C2M34 (232 vs. 226 bp) and C3M69 (134 vs. 149 bp) (Table 1; *14*). On the basis of these results, we postulated that the isolates from Tumbes might be related to clonet B. Surveys and available patient records revealed that 7 malaria cases occurred among members of a military facility in Tumbes. At least 1 of these patients reported having traveled to Yurimaguas, a small town in the Peruvian Amazon ≈400 km southwest of the city of Iquitos. For this reason, we compared the genotype of the parasites from Tumbes with those from 23 samples collected during 2009–2010 from Yurimaguas (12 samples) and from the Requena District (11 samples), which is 250 km northeast of Yurimaguas in the Peruvian Amazon (Figure 1). For all 11 isolates from Requena and 1 from Yurimaguas, the microsatellite genotypes were identical to that of the isolates from Tumbes—we named this parasite B_V1_ variant (Table 1).

To gain further insight into the drug-resistance pattern of the *P. falciparum* B_V1_ variant, we performed DNA sequencing to characterize point mutations in *Pfdhfr*, *Pfdhps*, *Pfcrt*, and *Pfmdr1,* which have been associated with resistance to sulfadoxine/pyrimethamine and chloroquine. Similar to clonet B, all outbreak samples shared 437/540/581 *Pfdhps*, SVMNT *Pfcrt*, and 184/1034/1042/1246 *Pfmdr1* genotypes. However, clonet B and the B_V1_ lineage differed in *Pfdhfr* haplotype. Whereas clonet B was 51I/108N/164L, the B_V1_ outbreak lineage and parasite isolate from Requena had the 50R/51I/108N *Pfdhfr* haplotype (Table 2; *14*). Typing of microsatellite loci near the boundaries of the *Pfdhfr* and *Pfmdr1* genes showed that the B_v1_ variant belonged to the haplogroup DHFR-D1, which in a previous study was found to be a rare haplogroup in Iquitos in 2006–2007 (only found in 3 [5%] of 62 samples); and MDR-A1, which in a previous study was found to be abundant in Iquitos in 2006–2007 (frequency >50%) (*16*).

**Table 2:** Drug resistant allele haplotypes of representative *Plasmodium falciparum* isolates from the Tumbes outbreak, clonets A–E, and isolates from the Amazon region, Peru, 2010–2012*

Code	Collection date	Source	Gene haplotypes				
			*Pfdhfr*	*Pfcrt*	*Pfdhps*	*Pfmdr1*	*Pfhrp2*
MIS 0760	2010 Oct 25	Tumbes	50R/51I/108N	SVMNT	437G/540E/581G	184F/1034C/1042D/1246Y	Neg
MIS 0761	2010 Oct 26	Tumbes	50R/51I/108N	SVMNT	437G/540E/581G	184F/1034C/1042D/1246Y	Neg
MIS 1142	2011 Mar 31	Tumbes	50R/51I/108N	SVMNT	437G/540E/581G	184F/1034C/1042D/1246Y	Neg
MIS 1147	2011 Jul 19	Tumbes	50R/51I/108N	SVMNT	437G/540E/581G	184F/1034C/1042D/1246Y	Neg
MIS 1146	2011 Jul 26	Tumbes	50R/51I/108N	SVMNT	437G/540E/581G	184F/1034C/1042D/1246Y	Neg
MIS 1168	2011 Aug 19	Tumbes	50R/51I/108N	SVMNT	437G/540E/581G	184F/1034C/1042D/1246Y	Neg
MIS1280	2011 Aug 27	Tumbes	50R/51I/108N	SVMNT	437G/540E/581G	184F/1034C/1042D/1246Y	Neg
TI-435	2011 Sep 22	Tumbes	50R/51I/108N	SVMNT	437G/540E/581G	184F/1034C/1042D/1246Y	Neg
TIA-4156	2011 Sep 28	Tumbes	50R/51I/108N	SVMNT	437G/540E/581G	184F/1034C/1042D/1246Y	Neg
T3–861	2011 Oct 4t	Tumbes	50R/51I/108N	SVMNT	437G/540E/581G	184F/1034C/1042D/1246Y	Neg
TIA-4333	2011 Oct 14t	Tumbes	50R/51I/108N	SVMNT	437G/540E/581G	184F/1034C/1042D/1246Y	Neg
MIS1278	2011 Nov 1	Tumbes	50R/51I/108N	SVMNT	437G/540E/581G	184F/1034C/1042D/1246Y	Neg
T3–989	2011 Nov 18	Tumbes	50R/51I/108N	SVMNT	437G/540E/581G	184F/1034C/1042D/1246Y	Neg
M01L1615	2011 Dec 1	Tumbes	50R/51I/108N	SVMNT	437G/540E/581G	184F/1034C/1042D/1246Y	Neg
TIA-434	2011 Dec 1	Tumbes	50R/51I/108N	SVMNT	437G/540E/581G	184F/1034C/1042D/1246Y	Neg
PSSI-205	2011 Dec 27	Corrales	50R/51I/108N	SVMNT	437G/540E/581G	184F/1034C/1042D/1246Y	Neg
Z1–22	2012 Mar 14	Zarumilla	50R/51I/108N	SVMNT	437G/540E/581G	184F/1034C/1042D/1246Y	Neg
PR-12	2009 Dec 17	Requena	50R/51I/108N	SVMNT	437G/540E/581G	184F/1034C/1042D/1246Y	Neg
PY013	2010 Mar	Yurimaguas	ND	ND	ND	ND	Neg
Clonet B	1999–2000	Loreto (E)	51I/108N/164I	SVMNT	437G/581G	184F/1042D and 184F/1034C/1042D/1246Y	4/18
Clonet A	1999–2000	Loreto (E)	51I/108N/164I	SVMNT	WT, 437G/581G, and 437G/540E/581G	184F/1034C/1042D and 184F/1034C/1042D/1246Y	1/24
Clonet C	1999–2000	Loreto (E, W)	108N	CVMNT	WT	184F/1034C/1042D	5/25
Clonet D	1999–2000	Loreto (E, W)	108N	CVMNT	540-SYN	184F/1034C/1042D and 184F/1034C/1042D/1246Y	9/15
Clonet E	1999–2000	Zarumilla	WT and 108N	CVMNT	WT	ND	Pos

*Only representative alleles are included in this table. Gene haplotypes are indicated by numbers where a mutated amino acid was found. C, central Amazon; E, eastern Amazon; W, western Amazon; neg, *Pfhrp2* negative; pos, *Pfhrp2* positive, 1/24, 1 of 24 isolates positive for *Pfhrp2; *ND, not determined; WT, wild type.

Because 40% of *P. falciparum* isolates from the Peruvian Amazon have major deletions of the genes coding for HRP2 and HRP3 (*25*), the most commonly used targets of rapid diagnostic tests (*25*), we investigated the presence of these deletions in these samples. All isolates in this outbreak lacked the *Pfhrp2* gene (Table 2).

## Discussion

Our molecular epidemiology investigation identified a possible source of the *P. falciparum* parasites causing a major outbreak of malaria in Tumbes, Peru, a region that had been free of falciparum malaria since 2006. Our results suggested that this outbreak was caused by a single introduction of a parasite population that originated in the Loreto region of the Peruvian Amazon. These parasites had a chloroquine- and sulfadoxine/pyrimethamine–resistant mutation pattern. This study illustrates the value of molecular epidemiology tools during malaria outbreak investigations and malaria reintroduction events.

All *P. falciparum* samples available for testing belonged to a single genetic lineage, according to their nearly identical microsatellite genotypes. For 2 isolates, 2 alleles were slightly different sizes (169 vs. 172 bp). This variation could represent microevolution of parasites during this outbreak, which has been observed during outbreaks involving other microsporidia parasites (*26*).

Because one of the first patients reported in October 2010 in Tumbes had a history of travel to Loreto, we hypothesized that the parasite population causing this outbreak could be associated with parasites from Loreto. The genotype of the parasites from Tumbes was identical to that of 11 of 11 *P. falciparum* isolates collected from the Requena District in Loreto in 2010 and 1 of 12 *P. falciparum* isolates collected from Yurimaguas (V. Udhayakumar, pers. comm.). Further analysis revealed that the Tumbes genotype was closely related to clonet B, which we previously reported to have been introduced to Loreto from Brazil before the major malaria epidemic in the 1990s (*14*,*16*). Furthermore, the parasite from Tumbes was highly unrelated to clonet E, which was the only parasite population found in the northern coast of Peru before falciparum malaria was eliminated in this region (*14*). These results demonstrate that the outbreak of falciparum malaria in Tumbes was most likely caused by a single event, the introduction of a parasite population from Loreto.

Consistent with the neutral microsatellite findings, all other genetic markers tested supported the aforementioned conclusion. Included were the chloroquine-resistant SVMNT genotype, pyrimethamine-resistant genotype (*Pfdhfr* 50R, 51I, and 108N), sulfadoxine-resistant genotype (437G, 540E, and 581G), and deletion of the *Pfhrp2* gene.

Several observations are relevant to the recent resurgence of malaria in Tumbes. First, this lineage has a new *Pfdhfr* triple mutant genotype (50R, 51I, 108N) that was mainly found outside of Peru in South America (*14*,*16*,*21*,*27*). This genotype seems to have been introduced into Peru during or after peak transmission of malaria within Peru because such genotypes were only reported in, not before, 2006 (*14*,*16*). Second, in the northern coast of Peru, artesunate–sulfadoxine/pyrimethamine combination therapy was being used for the treatment of *P. falciparum* malaria, while this newly introduced parasite strain had mutation patterns consistent with resistance to chloroquine and sulfadoxine/pyrimethamine. Coincidentally, malaria cases reported during this outbreak were treated with artesunate and mefloquine to decrease the risk for treatment failure. Therefore, the use of artesunate–sulfadoxine/pyrimethamine for the treatment of *P. falciparum* in the northern coast of Peru, as is currently recommended by treatment guidelines published by the Ministry of Health of Peru, may not be appropriate in this region because of the risk for malaria reintroduction from the Amazon region. Third, a region with no documented evidence of *Pfhrp2*-deleted parasites suddenly became populated with such a parasite strain, thereby making HRP2-based rapid tests inadequate diagnostic tools for this investigation. Misdiagnosis could have occurred if HRP2-based rapid tests were used as the primary diagnostic tool in this region.

In summary, this study provides experimental evidence of the value of timely molecular epidemiology investigations for pinpointing the source of *P. falciparum* reintroduction in areas working toward malaria elimination. Additionally, these data point out that future screening of military recruits (or any other migrant population from malaria-endemic areas such as the Amazon region) for the presence of malaria and provision of appropriate treatment can help prevent future reintroduction of malaria in areas from which it had been eliminated.
